# Interchangeability of different COVID-19 vaccine platforms as booster doses: A phase 3 study mimicking real-world practice

**DOI:** 10.1016/j.vaccine.2024.05.009

**Published:** 2024-07-25

**Authors:** Sue Ann Costa Clemens, Lily Weckx, Eveline P. Milan, Igor Smolenov, Ralf Clemens

**Affiliations:** a Institute for Global Health, University of Siena, Siena, Italy; b Department of Pediatrics, Oxford University, Oxford, UK; c CRIE UNIFESP, Reference Center for Special Immunobiologicals, Federal University of São Paulo, São Paulo, Brazil; d Centro de Estudos e Pesquisa em Moléstias Infecciosas Ltda. (CEPCLIN), Natal, Brazil; e Clover Biopharmaceuticals, Chengdu, China; f International Vaccine Institute IVI BOT, Seoul, Republic of Korea

**Keywords:** SARS-CoV-2, Protein vaccine, mRNA vaccine, Vector vaccine

## Abstract

**Background:**

The COVID-19 pandemic is over but the highly immunized or naturally exposed global population still requires booster vaccinations against newly emerging SARS-CoV-2 variants. We assessed safety and immunogenicity of booster doses of COVID-19 vaccines based on three different platforms in a setting that mimics the current routine practice in Brazil.

**Methods:**

In this phase 3 study from 14 February 2023 to 12 June 2023 we enrolled previously immunized adults to receive an additional booster dose of one of three vaccines. Immunogenicity against ancestor SARS-CoV-2 and Omicron BF.7, BQ.1.1.3, and XBB.1.5.6 sub-lineages was measured as ELISA IgG or virus neutralizing (VNT) antibodies and safety/reactogenicity assessed using diary cards.

**Results:**

Volunteers with a history of full primary COVID-19 immunization striated to three cohorts according to their previous booster vaccination history—0 (n = 26), 1 (n = 140) or 2 (n = 606) booster vaccinations—were randomized 2:1:1 to receive either recombinant protein (SCB-2019, Clover), adenovirus-vector (ChAdOx1-S, AstraZeneca/Fiocruz), or mRNA (BNT162b2, Pfizer/Wyeth). Baseline antibody titers were higher in individuals who had received one or two boosters and titers against both ancestor and Omicron sub-lineages increased in all groups regardless of the number of previous booster doses or the vaccine used. Day 28 geometric mean titers (GMTs) and geometric mean-fold rises (GMFR) against all variants were higher after BNT162b than SCB-2019 or ChAdOx1-S, but BNT162b groups displayed more rapid antibody waning at Day 84. Within cohorts each vaccine elicited similar GMFR against the different SARS-CoV-2 strains. All vaccines were well tolerated with similar solicited reactogenicity profiles.

**Conclusions:**

Protein, adenovirus-vector or mRNA vaccine boosters were equally well tolerated and immunogenic against ancestor SARS-CoV-2 and Omicron sub-lineages in fully primed adults with 0–2 prior boosters. BNT162b induced the highest immune responses but also the most rapid waning of antibodies 3 months after vaccination.

**Clinical Trial Registration:**ClinicalTrials.gov, identifier NCT05812586.

## Highlights

• Booster vaccinations are necessary to protect against emerging SARS-CoV-2 variants.
• We tested three vaccine platforms, mRNA, adenovirus-vector and recombinant protein.
• Using different vaccine platforms did not affect booster safety or immunogenicity.
• All three vaccines increased immunity against Omicron BF.7, BQ.1.1.3, and XBB.1.5.6.
• Fold-increases were similar for ancestor and Omicron variants within a platform.

## 1 Introduction

Although the global COVID-19 pandemic is no longer considered to be a Public Health Emergency of International Concern (PHEIC) by the WHO [1], the continuing emergence of new SARS-CoV-2 variants with increasing ability to evade vaccine-induced immunity means immunization efforts must continue [2]. Such efforts may be improved by use of new vaccine compositions targeting the novel variants as well as heterologous vaccination with different types of vaccine, which has been shown to increase effectiveness against the Omicron variant compared with primary course schedules and homologous boosters [3]. As the global epidemiology has changed, with high levels of immunity due to prior immunization, previous infection or a combination of both (hybrid immunity) [4], it is important to know the impact of heterologous vaccination and immunization history on the immune response. In particular, a protein based vaccine the development of which was funded by CEPI was included to generate additional heterologous booster safety and immunological data for a well-proven, scalable and low cost platform. This was reflected in the study design, where we used a 2:1:1 randomization. The availability of a protein-based COVID-19 vaccine was considered important to offer a potential alternative to overcome vaccine hesitancy due to the fear of adverse vaccine reactions following mRNA or vector-based COVID-19 vaccines.

Following the extensive immunization campaigns during the COVID-19 pandemic period we explored the safety, reactogenicity and immunogenicity of booster doses of different vaccine platforms in fully primed individuals with different SARS-CoV-2 vaccination backgrounds, including the number of booster doses received prior to their enrolment in this study. This would mimic the “real world scenario” at vaccination centers where individuals with different previous vaccination schemes present themselves for a booster dose. It is important to understand whether vaccine boosting, independent of the immunization status, will be effective with respect to immunogenicity against the new variants which continue to emerge.

The present study was conducted in Brazil to compare booster responses to three COVID-19 vaccines, the mRNA (BNT162b2, Pfizer/Wyeth) and adenovirus-vector (ChAdOx1-S, AstraZeneca/Fiocruz) vaccines registered in Brazil, and an adjuvanted protein-based vaccine candidate (SCB-2019, Clover) which has an emergency use authorization (EUA) in China. SCB-2019 has been shown to be highly immunogenic [5], [6], [7] and has the advantage of being manufactured using a known and licensed technology that can produce high quantities of vaccine at reasonable cost. Furthermore, the safety/reactogenicity profile of a booster dose of SCB-2019 seems to be better than mRNA or vectored vaccines while still producing protective immunity including higher neutralizing antibodies titers against the omicron variant than an inactivated vaccine [8].

## 2 Materials and methods

### 2.1 Trial design and participants

This was a phase 3, randomized, single-blind, multi-center study. Following approval by the site ethical review committees, the Brazilian National Ethical Committee and the Oxford Tropical Research Ethics Committee, 10.13039/501100000769Oxford University, UK the study protocol was registered on ClinicalTrials.gov, identifier NCT05812586. The study was done according to the ethical principles of the latest Declaration of Helsinki and the Council for International Organizations of Medical Sciences International ethical guidelines and ICH GCP guidelines. All participants signed an informed consent form at enrolment. A data safety monitoring board (DSMB) composed of independent vaccine experts reviewed the safety data and study integrity.

Eligible participants were healthy individuals aged 18 years or older who had been fully primed with of one of the licensed SARS-CoV-2 vaccines available in Brazil − two doses of ChAdOx1-S (AstraZeneca/Fiocruz), BNT162b2 (Pfizer/Wyeth), or Sinovac (Instituto Butantan), or one dose of Jcovden (Janssen) − and had then received either 0, 1, or 2 booster doses with the last at least four months previously. The main exclusion criteria were self-reported COVID-19 infection confirmed by RT-PCR or lateral flow test within the 4 weeks before enrolment, pregnancy, breastfeeding, or a history of severe adverse or allergic reaction to any of the study vaccines. A full list of protocol-defined inclusion and exclusion criteria is included in Supplementary material.

At enrolment participants were allocated to one of three study cohorts (A, B and C) according to the number of their previous boosters (0, 1, or 2) and each cohort was then randomized 2:1:1 using an interactive schedule to three groups to receive one booster dose of either SCB-2019, ChAdOx1-S or BNT162b2. Vaccines were masked to ensure study blind of the participants, and unblinded study nurses who administered the vaccines played no further role in the study; participants and laboratory personnel were blinded to vaccine administered.

### 2.2 Schedule of activities

On day 0, a baseline blood sample was taken before administration of the assigned booster vaccine. Participants were monitored for 30 min for immediate reactions, and then completed study diaries soliciting local (injection site pain, redness and swelling) and systemic (fatigue, headache, myalgia, arthralgia, loss of appetite, nausea, chills, and fever) adverse events for 7 days. Participants were contacted by telephone on Day 7 to ensure completion of the diary card. At subsequent visits on days 28 and 84 further blood samples were taken, and safety assessed by the investigator based on the completed diary cards and interview. Suspected cases of SARS-CoV-2 infection occurring during the study were confirmed by reverse-transcription polymerase chain reaction [RT-PCR] or lateral flow test.

### 2.3 Vaccines

One dose of SCB-2019 vaccine (Clover Biopharmaceuticals, Changxing, China) contains 30 μg SCB-2019 recombinant protein adjuvanted with 1.50 mg of the toll-like receptor agonist, CpG-1018 (Dynavax Technologies, Emeryville, CA, USA), and 0.75 mg aluminum hydroxide (Thousand Oaks Biopharmaceuticals, USA) in a volume of 0.5 mL for intramuscular (im) injection. The recombinant vaccine, ChAdOx1-S (AstraZeneca/Fiocruz), contains chimpanzee adenovirus codifying the SARS-CoV-2 Spike glycoprotein, each dose being in 0.5 mL for im injection. The mRNA vaccine, BNT162b2 (Pfizer/Wyeth) contains 30 μg mRNA coding for the SARS-CoV-2 Spike glycoprotein in a volume of 0.3 mL for im injection.

### 2.4 Immunogenicity

Sera were prepared immediately on Days 0, 28 and 84 and sent to the central laboratory (VisMederi, Siena, Italy) for immunogenicity testing. The primary immunogenicity endpoints were the titers of anti-S-protein IgG antibodies against the ancestor strain (Wuhan-Hu-1) and Omicron BA.5 sub-lineage at Day 28 measured by ELISA. Secondary endpoints were wild-type virus neutralizing antibody titers (VNT) measured in a micro-neutralization assay [9] against ancestor strain and Omicron BA.5, BF.7, BQ.1.1.3 and XBB.1.5.6 sub-lineages performed in subsets of participants from each group. Titers were calculated as the reciprocal of the highest dilution of the sample that prevents a cytopathic effect (MN_50_) and converted to IU/mL for ancestral strain by comparison with a WHO international standard 20/136 from the NIBSC.

### 2.5 Safety objectives

The primary safety objective was the incidence of serious adverse events (SAEs), adverse events of special interest (AESIs) and severe unsolicited adverse events (AEs), presented by number of previous boosters and vaccine platform throughout the study. A secondary objective was the reactogenicity during days 0–7, assessed as the incidence of solicited local (pain, redness and swelling at the injection site) and systemic (fatigue, headache, myalgia, arthralgia, loss of appetite, nausea, chills, and fever) adverse events on diary cards.

### 2.6 Statistical analysis

There was no formal statistical hypothesis, the sample size being based on the ability to detect at least one SAE in 1:120 participants who had received 2 previous boosters, and additionally, to recruit a sufficient number of participants with 0 or 1 booster to allow a descriptive summary of the safety and immunogenicity of booster vaccinations. The primary study immunogenicity objective was to assess the immune response as the increase in anti-Spike ELISA IgG antibodies at day 28: persistence of these responses at day 84 was a secondary objective. Titers were expressed as group geometric mean titers (GMT) and geometric mean-fold ratios (GMFR) compared with the baseline titer, calculated on the mean of the log-transformed value (for GMFR, on the difference of the logarithmically transformed data), followed by the anti-log transfer of the mean to express the results on the original scale. Two-sided 95 % confidence intervals (CI) for log GMT were obtained by the Least-Squares Means (LSMeans) based on the log-transformed data, and then anti-log transforming the confidence limits to obtain the CI for GMT. Other secondary immunogenicity objectives were the immune responses as VNT against the ancestral strain and Omicron BQ1.1.3, BF.7 and XBB1.5.6 SARS-CoV-2 sub-lineages at Days 28 and 84. The primary safety objective was the descriptive assessment of the occurrence of serious or severe adverse events throughout the study period, the local and systemic reactogenicity being a secondary safety objective.

Continuous variables were expressed as mean ± standard deviation (SD) and discrete/counting variables as median, quartiles 1 and 3. Categorical variables were presented as counts and percentages. Pairwise comparisons used the Student *t* test (paired within groups), with p < 0.05 considered significant. SAS Viya 4 was used for analyses.

### 2.7 Role of the funder

The funders had no involvement in data acquisition, analysis or preparation of the manuscript.

## 3 Results

**Fig. 1:**
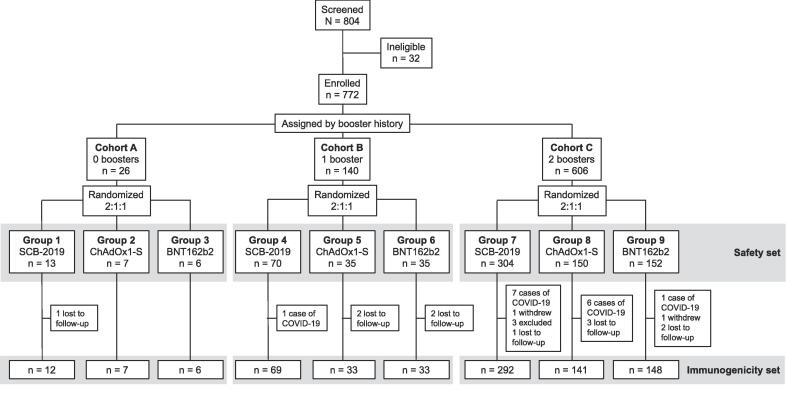
Study flow chart.

The study was performed from 14 February 2023 to 21 July 2023. From 804 individuals screened we enrolled 772 participants aged from 18 to 84 years (median 42) who were allocated to the three cohorts based on booster history and then randomized to the three groups to receive one of the three study vaccines (Fig. 1). All 772 enrolled participants received a booster vaccination and were included in the Exposed set and Safety set, 755 were included in the Full-Analysis set (FAS) and 694 were included in the Per-Protocol set (PPS).

The baseline demographics were balanced between cohorts and vaccine groups (Table 1). Most participants were female (472 of 772, 61 %) and described themselves as white (501 of 772, 65 %), the remainder being described as black or Afro-American (76, 10 %), Asian (46, 6 %), or of unknown (mainly mixed) race (146, 19 %), with one described as indigenous and one as Other. The median time since their last vaccination was 256 days (range: 124–645) which varied between cohorts: Cohort A had received their last primary vaccination about 470 days previously with no booster, while Cohort B had their only booster 370–406 days earlier and Cohort C had their second booster 253 days earlier. A small majority of participants, 439 of 772 (57 %), reported having had a previous SARS-CoV-2 infection, 7 of 26 (27 %, [95 % CI: 11.6–47.8]), 84 of 140 (60 %, [51.4–68.2]) and 348 of 606 (57 %, [53.4–61.4]) with no, one or two previous boosters, respectively. There were proportionally fewer reported COVID-19 cases in cohort A, but the small number of participants in this cohort precludes any meaningful conclusions. There were no major differences between cohorts B and C and no differences between groups within each cohort. A total of 15 participants, most from the largest cohort C, were excluded from the immunogenicity analyses due to a confirmed COVID-19 infection during the study.

**Table 1:** Demographics of the whole study population by cohort and by group.

Cohort			**A: No previous booster**			**B: One previous booster**			**C: Two previous boosters**			***All***
Group Booster vaccine			**Group 1 SCB-2019**	**Group 2** ChAdOx1-S	**Group 3** BNT162b2	**Group 4 SCB-2019**	**Group 5** ChAdOx1-S	**Group 6** BNT162b2	**Group 7 SCB-2019**	**Group 8** ChAdOx1-S	**Group 9** BNT162b2	
		**N =**	13	7	6	70	35	35	304	150	152	*772*
**Age** (years)		**Median**	**24**	**25**	**43**	**37**	**27**	**31**	**44**	**45**	**43**	***42***
		(Q1, Q3)	(21–38)	(22–42)	(33–54)	(25–48)	(23–37)	(26–45)	(34–59)	(29–57)	(31–60)	*(29,55)*
**Female**		**n**	**4**	**3**	**2**	**46**	**18**	**16**	**198**	**95**	**90**	**472**
		(%)	(31)	(43)	(33)	(66)	(51)	(46)	(65)	(63)	(59)	(61)
**Race**	White	**n**	**5**	**2**	**2**	**40**	**20**	**18**	**216**	**101**	**97**	***501***
		(%)	(38)	(29)	(33)	(57)	(57)	(51)	(71)	(67)	(64)	*(65)*
	Black ^a^	**n**	**2**	**1**	**1**	**5**	**5**	**3**	**29**	**19**	**11**	***76***
		(%)	(15)	(14)	(17)	(7)	(14)	(9)	(10)	(13)	(7)	*(10)*
	Other ^b^	**n**	**6**	**4**	**3**	**25**	**10**	**14**	**59**	**30**	**44**	***195***
		(%)	(46)	(57)	(50)	(36)	(29)	(40)	(19)	(20)	(29)	*(25)*
**Previous COVID-19**		**n**	**4**	**2**	**1**	**44**	**20**	**20**	**167**	**100**	**81**	***439***
		(%)	(31)	(29)	(17)	(63)	(57)	(57)	(55)	(67)	(53)	*(57)*
**COVID-19 during study**		**n**	**0**	**0**	**0**	**1**	**0**	**0**	**7**	**6**	**1**	***15***
		(%)				(1)			(2)	(4)	(1)	*(2)*
**Days since last dose ^c^**	**Median**	**473**	**481**	**469**	**402**	**370**	**409**	**253**	**253**	**253**	***256***	
		(Q1, Q3)	(423, 511)	(443, 530)	(439, 621)	(297, 488)	(329, 449)	(332, 491)	(222, 267)	(220, 263)	(225, 267)	*(230, 322)*

a: Includes African-American, b: Other includes Yellow, Indigenous, unknown and refused to answer, c: Time from last vaccination received to enrolment.

### 3.1 Immunogenicity – ELISA IgG

**Fig. 2:**
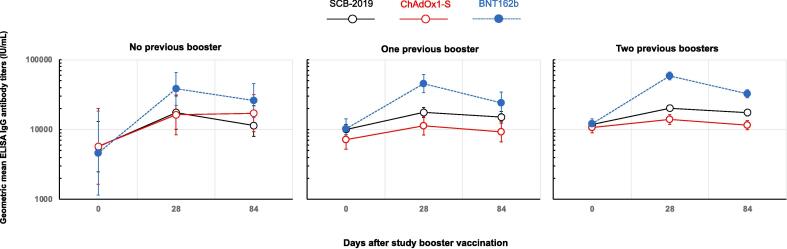
Geometric mean IgG titers (with 95% CI) against ancestor SARS-CoV-2 on Days 0, 28 and 84 after a booster vaccination with protein SCB-2019, adenovirus-vector ChAdOx1-S or mRNA BNT162b2 COVID-19 vaccines according to previous booster history (PPS excluding cases of COVID-19 during the study).

At baseline all participants had detectable IgG antibodies against the ancestral strain, the impact of the previous booster history on these baseline titers against the ancestral strain is shown in Table 2. Participants in Cohort A with no history of booster vaccination had lower titers than participants in Cohorts B and C who had received one or two boosters; the difference between Cohorts B and C was less than that between cohort A (no booster) and Cohort B (1 booster). In each cohort, baseline titers were generally similar across the three study groups (Table 3 and Fig. 2).

**Table 2:** Baseline IgG and virus neutralizing tiers (IU/mL) in the three different cohorts (FAS).

**SARS-CoV-2 strain**		**Cohort A** 0 boosters	**Cohort B** 1 booster	**Cohort C** 2 boosters
**ELISA IgG**				
	**N =**	**25**	**135**	**581**
**Ancestor**	**GMT**	**5917**	**9005**	**11589**
	(95 % CI)	(3758–9317)	(7863–10314)	(10689–12566)

**Virus neutralizing titers**				
**Ancestor**	**N =**	**25**	**88**	**140**
	**GMT**	**181**	**239**	**318**
	(95 % CI)	(107–308)	(198–289)	(268–376)
**Omicron XBB.1.5.6**	**GMT**	**28**	**40**	**46**
	(95 % CI)	(17–44)	(32–52)	(38–56)
**Omicron BQ.1.1.3**	**GMT**	**92**	**123**	**114**
	(95 % CI)	(48–174)	(94–160)	(94–139)
**Omicron BF.7**	**GMT**	**103**	**160**	**173**
	(95 % CI)	(57–186)	(124–206)	(142–212)

**Table 3:** Immune responses as IgG antibodies against Ancestor SARS-CoV-2 Spike protein.

		**No previous booster**			**One previous booster**			**Two previous boosters**		
		**Group 1**	**Group 2**	**Group 3**	**Group 4**	**Group 5**	**Group 6**	**Group 7**	**Group 8**	**Group 9**
		SCB-2019	ChAdOx1-S	BNT162b2	SCB-2019	ChAdOx1-S	BNT162b2	SCB-2019	ChAdOx1-S	BNT162b2
**Day**	**N =**	9	7	5	64	29	28	265*	134	139
**0**	**GMT (IU/mL)**	**5675**	**5738**	**4588**	**10021**	**7155**	**10200**	**11798**	**10662**	**12072**
	(95 % CI)	(2471–13036)	(1644–20028)	(1151–18285)	(8423–11924)	(5201–9844)	(7324–14206)	(10456–13311)	(8992–12641)	(10289–14164)

**28**	**GMT (IU/mL)**	**17485**	**16251**	**38314**	**17571**	**11283**	**45644**	**20277**	**13995**	**58771**
	(95 % CI)	(10081–30327)	(8454–31239)	(22129–66337)	(14904–20715)	(8361–15226)	(33939–61387)	(18363–22390)	(11857–16520)	(51840–66629)
	**GMFR**	3.08	2.83	8.35	1.75	1.58	4.47	1.72	1.31	4.87
	(95 % CI)	(0.89–10.7)	(0.87–9.24)	(1.72–40.54)	(1.47–2.10)	(1.29–1.93)	(3.06–6.55)	(1.57–1.88)	(1.19–1.45)	(4.20–5.64)

**84**	**GMT (IU/mL)**	**11398**	**17152**	**26114**	**15079**	**9301**	**24199**	**17484**	**11553**	**32565**
	(95 % CI)	(7967–21864)	(9336–31512)	(14906–45750)	(12490–18203)	(6686–12939)	(16992–34463)	(15666–19513)	(9912–13467)	(28626–37046)
	**GMFR vs Day 0**	2.33	2.99	5.69	1.50	1.30	2.37	1.48	1.08	2.70
	(95 % CI)	(0.81–6.66)	(1.30–6.89)	(1.73–18.8)	(1.23–1.83)	(0.97–1.74)	(1.72–3.27)	(1.33–1.64)	(0.96–1.22)	(2.34–3.10)
	**GMFR vs Day 28**	0.75	1.06	0.68	0.86	0.82	0.53	0.86	0.83	0.55
	(95 % CI)	(0.47–1.22)	(0.59–1.88)	(0.38–1.21)	(0.74–1.00)	(0.64–1.06)	(0.41–0.69)	(0.79–0.93)	(0.74–0.92)	(0.50–0.62)

* N = 264 at Day 84.

There were marked increases in IgG GMTs against ancestor strain in all groups when measured 28 days after a single dose of one of the three vaccines, achieving titers which were generally similar in magnitude for SCB-2019 and ChAdOx1-S vaccines but highest after the BNT162b2 vaccine. The highest geometric mean-fold ratios (GMFR) between Day 28 and baseline were observed in those with no previous history of booster vaccination and so the lowest baseline: 3.08 (95 % CI: 0.89–10.7) and 2.83 (0.87–9.24) for SCB-2019 and ChAdOx1-S, respectively, and 8.35 (1.72–40.5) for BNT162b2. GMFR were lower in the 1 and 2 booster dose cohorts, and lower after SCB-2019 and ChAdOx1-S than BNT162b2. This resulted in the highest GMTs being consistently observed in the mRNA vaccine boosted groups, and the lowest GMTs in the ChAdOx1-S vaccinees, with SCB-2019 GMTs generally being between the two (Table 3 and Fig. 2).

Waning of IgG antibodies by Day 84 is then demonstrated by the GMFR between Days 84 and 28 being less than 1 in all groups (except Group 2 which had a GMFR of 1.06 but consisted of only 7 participants). The rate of waning was greatest in the BNT162b2-boosted groups which had GMFRs of 0.53–0.68 between Days 84 and 28 when SCB-2019 and ChAdOx1-S groups had similar GMFRs of approximately 0.8, indicating a similar rate in the decline in titers. Nonetheless, with the highest post-booster responses the BNT162b2-boosted groups still had the highest GMTs for IgG antibodies at Day 84. For all groups, day 84 GMTs still exceeded the baseline GMTs, by a factor of 1·08 to 5·69.

The pattern of responses, as GMFR, was consistent when assessed on the basis of the last vaccine received, including ChAdOx1-S, BNT162b2 or Jcovden vaccines (Supplementary table 1). The GMFR at Day 28 were consistently highest with BNT162b2 as booster dose, and notably were higher when the primary series had been either ChAdOx1-S or Jcovden rather than BNT162b2. Highest rates of waning following BNT162b2 as booster were also evidenced by the lowest GMFR for Day84:Day28 in all groups.

### 3.2 Immunogenicity – Viral neutralizing titers

Baseline virus neutralizing titers also reflected the history of booster doses in the different cohorts, with a trend for GMTs against all tested SARS-CoV-2 strains to be higher in Cohort C after two boosters than Cohort B after one booster, which in turn were higher than Cohort A who had no history of previous booster vaccination (Table 2). This is confounded by the shorter interval since the last vaccination in Cohort C participants than either Cohorts A and B described above. However, differences between baseline GMTs were generally small, especially against the Omicron sub-lineages which had much lower titers than the ancestor strain, approximately half for Omicron BQ.1.13 and BF.7 sub-lineages and six-fold lower against Omicron XBB.1.5.6.

**Fig. 3:**
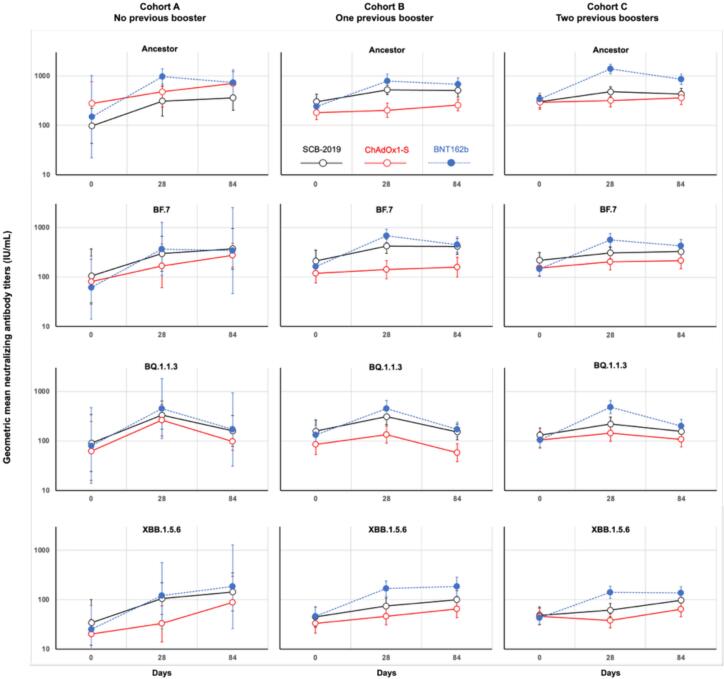
Geometric mean virus neutralizing titers (with 95% CI) against the indicated SARS-CoV-2 sub-lineages on Days 0, 28 and 84 after a booster vaccination with protein SCB-2019, adenovirus-vector ChAdOx1-S or mRNA BNT162b2 COVID-19 vaccines according to previous booster history (PPS excluding cases of COVID-19 during the study).

Fig. 3 illustrates how a booster dose of either of the three studied vaccines increased neutralizing GMTs against the SARS-CoV-2 ancestor and the three Omicron sub-lineages tested by Day 28 in all the different study groups (actual GMT values are presented in Supplementary table 2). Generally, the highest responses were observed in the BNT162b2 groups closely followed by the SCB-2019-boosted groups, with the lowest increases following a ChAdOx1-S booster. Notable exceptions to this were responses to SCB-2019 in Cohort C, who previously received two booster doses, in which SCB-2019 and ChAdOx1-S displayed similar profiles. The figure also shows that these increases were mostly transient with GMTs waning by Day 84, the notable exception being the responses to the Omicron XBB.1.5.6 sub-lineage and to a lesser extent the Omicron BF.7 sub-lineage.

As GMTs were influenced by the different baseline titers, the individual post-booster increases and eventual waning from Day 28 to Day 84 per group are shown as geometric mean-fold rises (GMFR) from baseline with statistical significance in Table 4**.** Generally, within each booster cohort for each vaccine platform the GMFR were similar against the different SARS-CoV-2 strains tested, although the low baseline levels of the later Omicron sub-lineages meant that final antibody GMTs were still low. Following BNT162b2 as booster there were statistically significant increases against each of the four tested strains in all three cohorts at Day 28, with the waning of these responses by Day 84 in most cases, shown as Day 84:Day 28 GMFR being less than 1, the exception being against XBB.1.5.6. Day 28:Day 0 increases were greatest in Cohort A for BTN162b2 vaccinees due to the lower baseline in this cohort with GMFR ranging from 4.92 to 6.50, compared with GMFR of 3.28–4.22 in Cohort B and 3.86–4.66 in Cohort C, but final GMTs according to booster vaccine were similar in all cohorts (Supplementary table 2*)*. Increases after SCB-2019 and ChAdOx1-S were lower in magnitude against all four strains in all three cohorts when compared with BNT162b2. With the exception of responses against Omicron BQ1.1.3, these increases persisted through to Day 84, with Day 84:Day 28 GMFR being almost 1 or greater. In contrast, the Day 84:Day 28 GMFR against Omicron BQ1.1.3 was less than 1 for all three vaccines showing the marked waning of this response.

**Table 4:** Geometric mean-fold ratios of neutralizing antibodies (95% CI) against Ancestor SARS-CoV-2 and Omicron sub-lineages.

		**Cohort A: No previous booster**			**Cohort B: One previous booster**			**Cohort C: Two previous boosters**		
		**Group 1**	**Group 2**	**Group 3**	**Group 4**	**Group 5**	**Group 6**	**Group 7**	**Group 8**	**Group 9**
**Strain**		SCB-2019	ChAdOx1-S	BNT162b2	SCB-2019	ChAdOx1-S	BNT162b2	SCB-2019	ChAdOx1-S	BNT162b2
	**N =**	9	7	5	29	27	26	43	44	48
**Ancestor**	**Day 28/Day 0**	**3.17**	**1.72**	**6.50**	**1.73**	**1.12**	**3.28**	**1.58**	**1.08**	**4.03**
	(95 % CI)	(0.92–11.0)	(0.67–4.42)	(1.35–31.2)	(1.26–2.39)	(0.88–1.43)	(2.30–4.67)	(1.28–1.95)	(0.90–1.29)	(3.13–5.18)
	***P***	*0.064*	*0.207*	***0.030***	***0.0015***	*0.338*	***<0.001***	***<0.0001***	*0.379*	***<0.0001***
	**Day 84/Day 28**	**1.17**	**1.49**	**0.76**	**0.95**	**1.28**	**0.90**	**0.90**	**1.13**	**0.62**
	(95 % CI)	(0.57–2.39)	(1.05–2.09)	(0.53–1.09)	(0.75–1.21)	(1.01–1.61)	(0.73–1.10)	(0.75–1.08)	(0.94–1.37)	(0.52–0.74)
	***P***	*0.634*	***0.030***	*0.099*	*0.674*	*0.042*	*0.276*	*0.242*	*0.192*	***<0.0001***

**Omicron BF.7**	**Day 28/Day 0**	**2.83**	**2.10**	**6.06**	**2.00**	**1.18**	**4.22**	**1.40**	**1.34**	**3.86**
	(95 % CI)	(1.00–8.02)	(0.62–7.11)	(1.99–18.5)	(1.34–2.97)	(0.95–1.47)	(2.95–6.03)	(1.06–1.86)	(1.09–1.64)	(2.79–5.33)
	***P***	***0.050***	*0.187*	***0.0109***	***0.0013***	*0.125*	***<0.0001***	***0.0197***	***0.0063***	***0.0013***
	**Day 84/Day 28**	**1.26**	**1.64**	**0.93**	**0.94**	**1.12**	**0.67**	**1.07**	**1.06**	**0.77**
	(95 % CI)	(0.43–3.69)	(0.61–4.43)	(0.21–4.19)	(0.75–1.18)	(0.86–1.46)	(0.55–0.81)	(0.89–1.27)	(0.82–1.36)	(0.65–0.90)
	***P***	*0.633*	*0.2698*	*0.905*	*0.583*	*0.375*	***0.0002***	*0.465*	*0.660*	***0.0013***

**Omicron BQ1.1.3**	**Day 28/Day 0**	**3.70**	**4.21**	**5.66**	**1.95**	**1.57**	**3.41**	**1.68**	**1.37**	**4.66**
	(95 % CI)	(0.90–15.2)	(1.22–14.5)	(2.53–12.7)	(1.24–3.07)	(1.23–2.00)	(2.45–4.74)	(1.36–2.06)	(1.10–1.71)	(3.42–6.33)
	***P***	*0.0645*	***0.0293***	***0.0040***	***0.0051***	***0.0009***	***<0.0001***	***<0.0001***	***0.0063***	***<0.0001***
	**Day 84/Day 28**	**0.48**	**0.37**	**0.38**	**0.48**	**0.43**	**0.38**	**0.71**	**0.74**	**0.41**
	(95 % CI)	(0.24–0.98)	(0.17–0.82)	(0.08–1.76)	(0.36–0.64)	(0.34–0.55)	(0.29–0.50)	(0.55–0.92)	(0.54–1.02)	(0.33–0.51)
	***P***	*0.0449*	***0.0225***	*0.154*	***<0.0001***	***<0.0001***	***<0.0001***	***0.0116***	***0.0634***	***<0.0001***

**Omicron XBB1.5.6**	**Day 28/Day 0**	**3.06**	**1.64**	**4.92**	**1.67**	**1.40**	**3.64**	**1.28**	**0.83**	**3.24**
	(95% CI)	(1.33–7.01)	(0.78–3.43)	(1.24–19.6)	(1.27–2.20)	(1.09–1.79)	(2.39–5.56)	(1.05–1.56)	(0.68–1.00)	(2.542–4.14)
	***P***	***0.0145***	*0.152*	***0.0327***	***0.0006***	***0.0110***	***<0.0001***	***0.0147***	*0.054*	***<0.0001***
	**Day 84/Day 28**	**1.36**	**2.69**	**1.52**	**1.31**	**1.41**	**1.15**	**1.58**	**1.70**	**0.98**
	(95% CI)	(0.53–3.47)	(0.69–10.6)	(0.17–13.4)	(1.02–1.70)	(1.12–1.79)	(0.90–1.47)	(1.32–1.90)	(1.31–2.20)	(0.85–1.13)
	***P***	*0.470*	*0.126*	*0.625*	***0.0386***	***0.0059***	*0.253*	***<0.0001***	***0.0002***	*0.763*

*P* values are for pair-wise comparisons of Day 28 & Day 0, or Day 84 & Day 28 values.

### 3.3 Safety

There were 7 SAEs (epileptic seizure, cholelithiasis, high digestive bleeding, acute myocardial infarction, appendicitis, ophthalmic herpes zoster, abortion) reported between 9 and 93 days after the booster dose among all participants who were vaccinated in the present study; 4 after SCB-2019 and 3 after BNT162b2. None of these events was considered to have a causal relationship with the vaccinations and all participants recovered without sequelae. There were no adverse events of special interest (AESI) reported. There were two pregnancies, both vaccinated with SCB-2019, one which resulted in a spontaneous abortion 75 days after vaccination, and a second which resulted in a full-term birth of a healthy child. Of 22 unsolicited grade 3 or 4 adverse events, one of was considered to have a possible temporal relationship with the vaccination, a gastrointestinal disorder 2 days after a booster dose of SCB-2019 (Supplementary table 3).

**Fig. 4:**
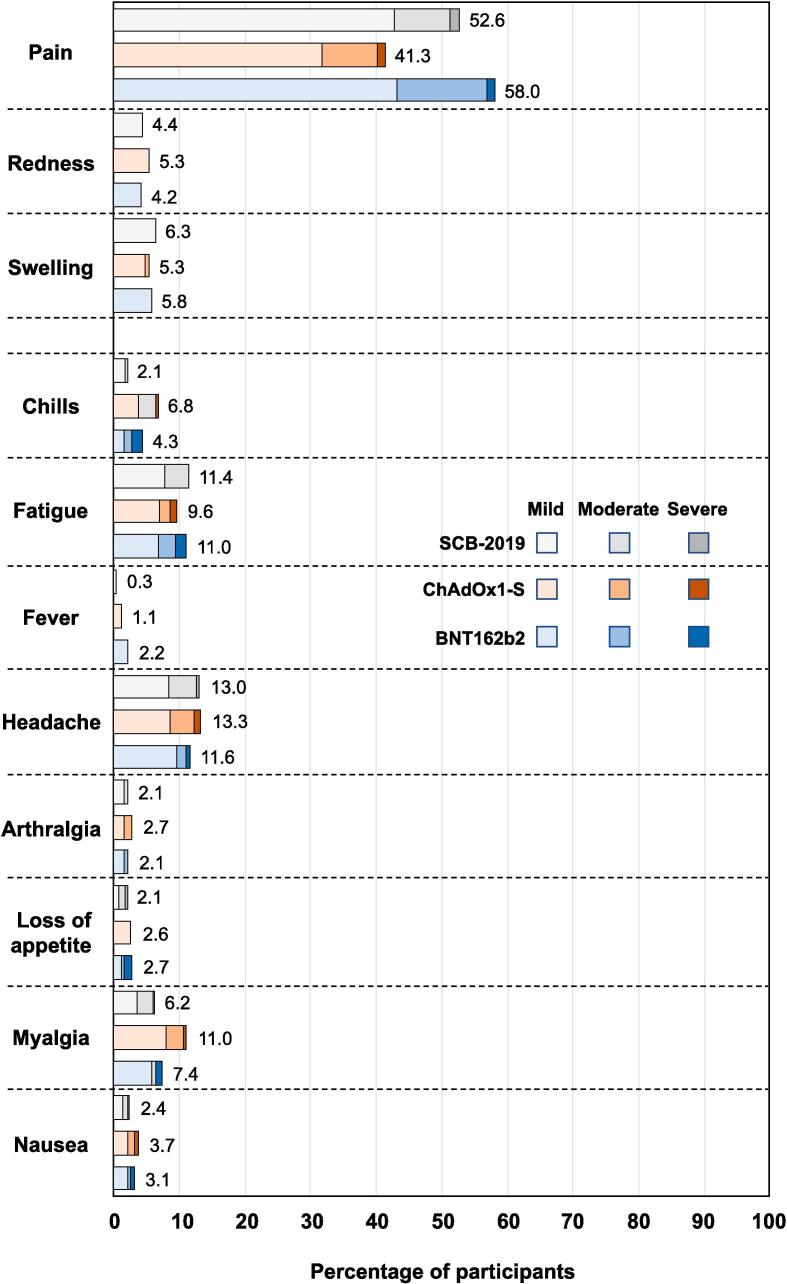
Reactogenicity by severity of the three booster vaccines across groups.

All three vaccines were generally well tolerated with similar solicited reactogenicity profiles (Fig. 4). The most frequent solicited adverse event was injection site pain, reported by 52.6 %, 41.3 % and 58.0 % of SCB-2019, ChAdOx1-s and BNT162b2 recipients. Only about 1.2 % of such reports were described as severe (Grade 3) and all were transient and resolved within the reporting period. The most frequent systemic adverse events were headache, in 13.0 %, 13.3 % and 11.6 %, and fatigue, in 11.4 %, 9.6 % and 11.0 %, of SCB-2019, ChAdOx1-S and BNT162b2 groups. There were no obvious trends to variations in these profiles across cohorts or according to previous vaccination history.

There were 15 participants who had a confirmed COVID-19 infection, 6 within 1 week of booster, 5 within 4 weeks of booster, and the rest thereafter. Of these, 8 (2.1 %) occurred in 387 SCB-2019 vaccine recipients, 6 (3.1 %) in 192 ChAdOx1-S recipients and 1 (0.5 %) in 193 BNT162b2 recipients (Table 1).

## 4 Discussion

The COVID-19 pandemic has abated, but continuing indications of seasonal increases in cases of COVID disease due to emerging variants requires ongoing immunization efforts [2]. We assessed the responses to a booster dose of three different COVID-19 vaccines based on the ancestral SARS-CoV-2 viral spike protein produced using different platforms in adults who previously received complete priming series of different vaccines with either no or 1 or 2 previous booster doses. All three vaccines were well tolerated and induced increases in IgG antibodies against the ancestral strain and neutralizing immunity against ancestral and Omicron sub-lineages, which were circulating during the study period. The mRNA vaccine, BNT162b2, induced higher responses in magnitude and fold-rise in titers than the adenovirus-vector vaccine, ChAdOx1-S and recombinant protein vaccine, SCB-2019, but displayed a more rapid rate of waning of both binding and neutralizing antibodies as previously reported [10], [11]. Responses and tolerability did not appear to be influenced either by the previous immunization history, the previous vaccine type or number of doses, suggesting healthcare providers can be confident that future immunization campaigns can use any suitable vaccine.

The study population had been highly exposed to SARS-CoV-2, 57 % reporting a previous COVID-19 infection, which would result in hybrid immunity [4]. In these circumstances it is important to know whether any combination of booster and priming vaccine and previous boosting history would raise any concerns about safety and increase in immunity. Such concerns override any questions about the actual magnitude of specific responses, as future immunization campaigns will be performed using new compositions of COVID vaccines targeting future SARS-CoV-2 variants rather than the ancestral Wuhan-Hu-1 strain used to design the first wave of vaccines.

It is reassuring, therefore, to note that the various combinations of protein-based, vector-based or mRNA booster vaccines used in the present study all resulted in increases in immunity with little or no difference in tolerability or safety including increases in neutralizing activity against some of the recent Omicron sub-lineages with vaccines designed against the ancestral strain. There were marked variations in the magnitude of these responses; overall, the mRNA vaccine consistently induced the highest titers four weeks after vaccination, but this was tempered by the observation that the mRNA response also displayed the most rapid waning of binding and neutralizing antibodies between 1- and 3-months post-vaccination. However, even though the rate of waning was highest after BNT162b2, IgG and neutralizing titers were still higher at day 84 after this vaccine compared with the other two vaccines for most analyses. We do not have data on whether this decline persisted at a higher rate after this last time point, but effectiveness of a mRNA vaccine booster against Omicron variants has previously been reported to fall to less than 20 % within 6 months of booster vaccination [12]. Responses to SCB-2019 were generally higher than those to ChAdOx1-S, particularly in those who had previously received one or two booster vaccinations. SCB-2019 is a protein-based vaccine produced by an easily scalable technology that can provide vaccines for lower-income countries at an affordable price. As immune responses and the safety profile of SCB-2019 were similar, or even superior to that of ChAdOx1-S with regard to titers, this study supports the use of this platform for vaccines against future COVID outbreaks.

Our data show that all three vaccines increased neutralizing responses against more recent Omicron sub-lineages, although to much lower extents than against the original ancestral strain. With the inherent delays in vaccine development there will always be a lag period between vaccine design targeting a selected variant, and the actual variants in circulation by the time of vaccine implementation. However, the broad response we observed against new emergent variants is reassuring and may indicate an improved capacity to protect against severe disease. In another study, a monovalent mRNA vaccine updated to target the XBB.1.5 sub-lineage has been shown to induce 13.3-to-27.6-fold increases in neutralizing activity against more recent variants [13] including the JN.1 sub-lineage derived from Omicron BA.2.86, which now predominates globally [14]. It is anticipated therefore that booster vaccination with such a new vaccine composition will protect against the consequences of these latest variants as previously observed with complete primary and booster series of the original ancestral strain vaccines against the subsequent waves of Omicron sub sub-lineages [15].

Furthermore, the variety of vaccine platforms available will make it difficult to track exact immunization histories for individuals, especially if COVID-19 vaccination becomes a routine seasonal procedure, so it is important to observe from our data that mixing vaccines does not appear to impact the safety or tolerability of new booster vaccinations. mRNA vaccines have been associated with some serious or severe adverse events, most notably cardiac disorders [16]. BNT162b2 itself has been associated with a risk difference of 18.0 SAEs per 10,000 vaccinees [17], and in a Cochrane review the relative risk of SAEs for ChAdOx1-S in 58,182 vaccinees was 0.88 (95 % CI 0.72–1.07) and 1.30 (95 % CI 0.55–3.07) for 46,107 BNT162b2 vaccinees [18]. A safety review of SCB-2019 found no difference between the recombinant protein and placebo though based on much smaller numbers [19].

Our study has several limitations, not least in being restricted to three vaccines representing three different vaccine platforms, mRNA, viral-vector and protein-based, from the wide variety of different vaccines which were available during the pandemic. Nonetheless we believe these are sufficiently representative of the different types most widely available. As already noted, immunological responses were measured against the ancestral strain and some of the more common later Omicron sub-lineages, while future immunization campaigns will be done with new vaccine compositions using these vaccine platforms and targeting new strains which will require separate clinical testing. This will also necessitate assessment of other components of the immunological response including T cell activation and other aspects of humoral and cellular immunity, as well as the duration of the responses. The study was not designed to assess vaccine efficacy and the cases of COVID-19 reported during it were insufficient to draw any conclusions about effectiveness. However, a significant correlation has been demonstrated between the IgG-binding and neutralizing responses and clinical efficacy of BNT162b2 and ChAdOx1-S sufficient to justify our limited conclusions [20].

We looked at a real-world situation in which adults who had previously been immunized with a complete primary series of a variety of COVID-19 vaccines were then boosted with one of three different vaccines representing some of the most common vaccine platforms available. This showed that despite differences between vaccines in the initial immune responses all combinations induced increases in protective antibodies against all tested SARS-CoV-2 strains with no impact on safety or tolerability. As increases in neutralizing antibodies included those against some of the most recent Omicron sub-lineages this suggests that booster immunization campaigns can be implemented with whichever vaccines are available.

## Funding

This work was funded by the Instituto D'Or de Ensino e Pesquisa (IDOR) supported by a grant from the Bill & Melinda Gates Foundation (BMGF) [grant number INV-030336].

## CRediT authorship contribution statement

**Sue Ann Costa Clemens:** Writing – review & editing, Supervision, Investigation, Funding acquisition, Conceptualization. **Lily Weckx:** Writing – review & editing, Resources, Project administration, Investigation, Conceptualization. **Eveline P. Milan:** Writing – review & editing, Resources, Project administration, Investigation. **Igor Smolenov:** Writing – review & editing, Supervision, Project administration, Methodology, Investigation, Formal analysis, Data curation, Conceptualization. **Ralf Clemens:** Writing – review & editing, Writing – original draft, Supervision, Funding acquisition, Conceptualization.

## Declaration of competing interest

The authors declare the following financial interests/personal relationships which may be considered as potential competing interests: Sue Ann Costa Clemens reports financial support was provided by Bill & Melinda Gates Foundation. Igor Smolenov reports a relationship with Clover Pharmaceuticals Inc. that includes: employment and equity or stocks. Ralf Clemens reports a relationship with Clover Pharmaceuticals Inc. that includes: board membership. Sue Ann Costa Clemens is a Professor in Global Health, Department of Paediatric Infectious Diseases, Oxford University, UK, Head of the Institute for Global Health, University of Siena, Italy, Director Vaccine Group Oxford-Brazil, and Senior Adviser at the Bill & Melinda Gates Foundation (BMGF) and member of the Scientific Advisory Board of various vaccine organizations including Clover Biopharmaceuticals Inc. Igor Smolenov was a full-time employee of Clover Biopharmaceuticals at the time of the study. Ralf Clemens is a Senior Advisor to BMGF and member of the Board of Trustees of the International Vaccines Institute (IVI) and member or chair of various vaccine companies including Clover Biopharmaceuticals Inc. Eveline P. Milan and Lily Weckx have no conflicts to declare. If there are other authors, they declare that they have no known competing financial interests or personal relationships that could have appeared to influence the work reported in this paper.

## Data Availability

The data that support the findings of this study are available on request from the corresponding author, SCC.
