# Supplementary material for: Interchangeability of different COVID-19 vaccine platforms as booster doses: A phase 3 study mimicking real-world practice
Source: Vaccine. 2024 Jul 25;42(19):3989–98. doi: 10.1016/j.vaccine.2024.05.009 (PMC11252665; doi:10.1016/j.vaccine.2024.05.009)
Supplement: Supplementary Data 1 [file mmc1.docx]

**Supplementary material**

**Costa Clemens et al**

**Interchangeability of various COVID-19 vaccine platforms as booster doses: a phase 3 study mimicking real-world practice**

**Inclusion criteria** *page 2*

**Exclusion criteria** *pages 3-4*

**Table 1:** GMFRs of IgG antibody responses *page* *5*

**Table 2:** GMTs of virus neutralizing antibody responses *pages 6-7*

**Table 3:** Grade 3 and 4 adverse events *page* *8*

**Inclusion criteria**

Participants were eligible to be included in the study only if all of the following criteria applied:

1. Male or female ≥18 years of age.

2. Individuals were willing and able to comply with study requirements, including all scheduled visits, vaccination, laboratory tests, and other study procedures.

3. Individuals were willing and able to give an informed consent, prior to screening.

4. Individuals must have completed vaccine priming, regardless of vaccine regimen. Primary vaccination and previous booster scheme data were be annotated as patient history.

5. Interval between last dose and study dose of a minimum of 4 months and a maximum of 24 months (to optimize candidate participation).

6. Healthy participants or participants with pre-existing medical conditions who were in a stable medical condition. A stable medical condition was defined as disease not requiring significant change in therapy or hospitalization for worsening disease during the 3 months before enrolment.

7. Female participants were eligible to participate in the study if not pregnant, not breastfeeding, and at least 1 of the following criteria applied:

• Women of non-childbearing potential;

• Women of childbearing potential (WOCBP) must have had a negative urine pregnancy test prior to study vaccination. A confirmatory serum pregnancy test may have been conducted at the investigator’s discretion. They must have been using a highly effective licensed method of birth control during the study, until 90 days after the study vaccination.

**Exclusion criteria**

Participants were excluded from the study if any of the following criteria applied:

1. Individuals with fever >37.5°C (axillary), or any acute illness at baseline (Day 0) or within 3 days prior to randomization. Participants meeting this criterion may have been rescheduled within the relevant window. Febrile participants with minor illnesses could be enrolled at the discretion of the investigator.
2. Self-reported confirmed COVID-19 infection, through RT-PCR or lateral flow test, in the last 4 weeks.
3. Individuals who did not complete the primary vaccination scheme for any licensed or experimental COVID vaccine or planned to receive another COVID-19 vaccine (other than the study vaccines) during the study period, a drug for COVID-19 prevention or treatment (e.g., drugs, monoclonal antibodies, such as Rituximab or any other anti-CD20 monoclonal antibodies during the study period.).
4. Individuals who had a history of severe adverse reaction associated with a vaccine or severe allergic reaction (e.g., anaphylaxis, venal or arterial thrombosis, thrombocytopenia) to any component of the study vaccines (Pfizer/Wyeth, AstraZeneca/Fiocruz, CpG 1018, aluminum, or SCB-2019 components, as outlined in the latest summary of product characteristics for Pfizer/Wyeth, AstraZeneca/Fiocruz, and the IB for SCB-2019/Clover).
5. Individuals with capillary leakage syndrome or thrombosis with thrombocytopenia syndrome – TTS (possibly associated with vaccination with the AstraZeneca/Fiocruz vaccine).
6. Individuals who had pericarditis or myocarditis (these pathologies may be associated with the Pfizer/Wyeth vaccine, especially in young men).
7. Individuals with a known bleeding disorder that, in the opinion of the investigator, contraindicated intramuscular injection.
8. Individuals who had a history of malignancy within 1 year before screening (exceptions were squamous and basal cell carcinomas of the skin and carcinoma in situ of the cervix which had been cured, or other malignancies with minimal risk of recurrence).
9. Individuals with any progressive or severe neurologic disorder, seizure disorder, or history of Guillain-Barré syndrome.
10. Individuals who received treatment with immunosuppressive therapy in the last 90 days, including cytotoxic agents or systemic corticosteroids, or planned receipt during the study period. If a short-term course of systemic corticosteroid immunosuppressive dose had been used for the treatment of acute illness, the participant should not have been included in the study until corticosteroid therapy had been discontinued for at least 15 days prior to first study vaccination. If the participant had used an immunosuppressive dose of a depot corticosteroid, intra-muscular or intra-articular, they must had waited60 days for inclusion in the study. Inhaled/nebulized, intra-articular, intrabursal, or topical (skin or eyes) corticosteroids were permitted.
11. Individuals with autoimmune diseases, except: Hashimoto’s thyroiditis, vitiligo, psoriasis, lupus discord and alike; HIV-positive individuals and/or on HIV treatment.
12. Individuals who had received any other investigational product within 30 days prior to Day 0 or intended to participate in another clinical study at any time during the conduct of the study.
13. Individuals who had received any other licensed vaccines within 14 days prior to enrollment in the study or who were planning to receive any vaccine up to 28 days after the last vaccination.
14. Individuals who had received treatment with Rituximab or any other anti-CD20 monoclonal antibodies within 9 months prior to Day 0 or planned to receive this drug during the study period.
15. Individuals who received intravenous immunoglobulins and/or any blood products within 3 months prior to enrollment or planned administration during the study period.
16. Individuals with any condition that, in the opinion of the investigator, would interfere with the primary study objectives or pose additional risk to the participant.
17. Pregnancy.
18. Breastfeeding.

| **Supplementary table 1:** Geometric mean fold ratios (95% CI) of IgG antibodies against ancestor SARS-CoV-2 Spike protein according to previous vaccination history. | | | | | |
| --- | --- | --- | --- | --- | --- |
| **Previous vaccine** | **No. of boosters** | **GMFR** | **Present booster vaccine** | | |
|  |  |  | **SCB-2019** | **ChAdOx1-S** | **BNT162b2** |
| **ChAdOx1-S** | **1** | Day 28 / Day 0 | **2.10**  (1.47, 3.00) | **1.99**  (1.07, 3.67) | **6.60**  (3.49, 12.47) |
|  |  | Day 84 / Day 28 | **0.85**  (0.66, 1.09) | **0.67**  (0.53, 0.84) | **0.47**  (0.30, 0.72) |
|  |  | Day 84 / Day 0 | **1.78**  (1.33, 2.39) | **1.32**  (0.81, 2.16) | **3.09**  (1.63, 5.83) |
|  | **2** | Day 28 / Day 0 | **1.74**  (1.54, 1.97) | **1.11**  (0.99, 1.26) | **5.91**  (4.59, 7.62) |
|  |  | Day 84 / Day 28 | **0.88**  (0.78, 1.01) | **0.96**  (0.80, 1.15) | **0.51**  (0.42, 0.61) |
|  |  | Day 84 / Day 0 | **1.54**  (1.32, 1.80) | **1.07**  (0.88, 1.30) | **1.54**  (1.32, 1.80) |
| **BNT162b2** | **1** | Day 28 / Day 0 | **1.51**  (1.19, 1.93) | **1.42**  (1.12, 1.80) | **2.78**  (1.89, 4.09) |
|  |  | Day 84 / Day 28 | **0.80**  (0.64, 1.00) | **0.91**  (0.68, 1.22) | **0.62**  (0.42, 0.92) |
|  |  | Day 84 / Day 0 | **1.21**  (0.90, 1.63) | **1.29**  (0.89, 1.88) | **1.72**  (1.27, 2.33) |
|  | **2** | Day 28 / Day 0 | **1.42**  (1.18, 1.71) | **1.36**  (1.07, 1.73) | **3.86**  (2.66, 5.60) |
|  |  | Day 84 / Day 28 | **0.91**  (0.73, 1.12) | **0.84**  (0.65, 1.08) | **0.50**  (0.38, 0.67) |
|  |  | Day 84 / Day 0 | **1.28**  (0.98, 1.67) | **1.14**  (0.77, 1.68) | **1.95**  (1.32, 2.88) |
| **Jcovden** | **2** | Day 28 / Day 0 | **1.82**  (1.54, 2.16) | **1.50**  (1.26, 1.79) | **4.56**  (3.65, 5.70) |
|  |  | Day 84 / Day 28 | **0.80**  (0.71, 0.91) | **0.72**  (0.62, 0.84) | **0.64**  (0.55, 0.74) |
|  |  | Day 84 / Day 0 | **1.46**  (1.21, 1.75) | **1.08**  (0.91, 1.29) | **1.46**  (1.21, 1.75) |

| **Supplementary table 2:** Neutralizing antibody geometric mean titers (95% CI) against Ancestor SARS-CoV-2 and Omicron sub-lineages | | | | | | | | | | |
| --- | --- | --- | --- | --- | --- | --- | --- | --- | --- | --- |
|  |  | **Cohort A: No previous booster** | | | **Cohort B: One previous booster** | | | **Cohort C: Two previous boosters** | | |
|  |  | **Group 1** | **Group 2** | **Group 3** | **Group 4** | **Group 5** | **Group 6** | **Group 7** | **Group 8** | **Group 9** |
| **Strain** |  | SCB-2019 | ChAdOx1-S | BNT162b2 | SCB-2019 | ChAdOx1-S | BNT162b2 | SCB-2019 | ChAdOx1-S | BNT162b2 |
|  | **N =** | 9 | 7 | 5 | 29 | 27 | 26 | 43 | 44 | 48 |
| **Ancestor** | **Day 0** | **97** | **276** | **149** | **301** | **180** | **239** | **300** | **293** | **341** |
|  | (95% CI) | (43–221) | (100–758) | (22–1008) | (213–427) | (130–249) | (164–348) | (227–396) | (210–410) | (258–452) |
|  | **Day 28** | **308** | **476** | **970** | **522** | **202** | **782** | **475** | **318** | **1376** |
|  | (95% CI) | (153–619) | (235–960) | (677–1390) | (422–646) | (145–280) | (565–1081) | (375–602) | (234–431) | (1103–1715) |
|  | **Day 84** | **359** | **707** | **735** | **506** | **257** | **676** | **428** | **360** | **854** |
|  | (95% CI) | (201–642) | (409–1220) | (413–1310) | (372–689) | (196–338) | (507–902) | (328–557) | (262–495) | (670–1089) |
| **Omicron BF.7** | **Day 0** | **105** | **80** | **61** | **211** | **119** | **162** | **221** | **153** | **147** |
|  | (95% CI) | (30–367) | (28–228) | (14–264) | (128–348) | (768–188) | (108–243) | (154–317) | (106–220) | (104–208) |
|  | **Day 28** | **296** | **168** | **368** | **421** | **141** | **684** | **310** | **204** | **566** |
|  | (95% CI) | (132–665) | (61–467) | (106–1270) | (301–590) | (92–215) | (502–932) | (237–406) | (140–298) | (417–769) |
|  | **Day 84** | **373** | **276** | **343** | **415** | **158** | **453** | **330** | **216** | **433** |
|  | (95% CI) | (159–4784) | (159–478) | (46–2545) | (289–597) | (100–249) | (317–646) | (248–441) | (149–312) | (325–578) |
| **Omicron BQ1.1.3** | **Day 0** | **90** | **62** | **80** | **158** | **85** | **133** | **131** | **105** | **104** |
|  | (95% CI) | (24–338) | (16–246) | (14–472) | (94–265) | (53–138) | (85–207) | (93–184) | (72–154) | (75–146) |
|  | **Day 28** | **333** | **263** | **453** | **309** | **134** | **453** | **219** | **144** | **486** |
|  | (95% CI) | (172–643) | (126–548) | (112–1825) | (214–445) | (90–198) | (309–663) | (158–305) | (98–214) | (358–661) |
|  | **Day 84** | **160** | **98** | **171** | **152** | **58** | **171** | **156** | **107** | **199** |
|  | (95% CI) | (78–328) | (65–147) | (31–944) | (106–219) | (38–88) | (122–240) | (123–199) | (76–151) | (146–271) |

| **Omicron XBB1.5.6** | **Day 0** | **34** | **20** | **25** | **45** | **33** | **46** | **48** | **46** | **43** |
| --- | --- | --- | --- | --- | --- | --- | --- | --- | --- | --- |
|  | (95% CI) | (12–100) | (10–40) | (8–76) | (28–715) | (21–52) | (29–752) | (218–71) | (31–67) | (32–58) |
|  | **Day 28** | **105** | **33** | **121** | **74** | **46** | **167** | **61** | **38** | **141** |
|  | (95% CI) | (0–219) | (14–75) | (26–562) | (51–109) | (31–695) | (116–239) | (45–84) | (27–52) | (106–186) |
|  | **Day 84** | **143** | **88** | **184** | **100** | **65** | **118** | **97** | **64** | **137** |
|  | (95% CI) | (59–345) | (26–296) | (268–1277) | (66–152) | (43–99) | (118–286) | (69–137) | (45–92) | (103–184) |
|  | | | | | | | | | | |

| **Supplementary table 3:** Unsolicited severe (Grade 3) and life-threatening (Grade 4) adverse events. | | | | | | |
| --- | --- | --- | --- | --- | --- | --- |
|  |  | **Grade 3** | | **Grade 4** | |  |
| **Symptom** | **Vaccine** | Not related | Related | Not related | Related | Total |
| Cardiac disorders | SCB-2019 | 0 | 0 | 1 | 0 | 1 |
| Ear and labyrinth disorders | BNT162b2 | 1 | 0 | 0 | 0 | 1 |
| Eye disorders | SCB-2019 | 1 | 0 | 0 | 0 | 1 |
| Food poisoning | SCB-2019 | 1 | 0 | 0 | 0 | 1 |
| Gastrointestinal disorders | SCB-2019 | 1 | 1 | 0 | 0 | 2 |
| General disorders and administration site conditions | SCB-2019 | 1 | 0 | 0 | 0 | 1 |
| Hepatobiliary disorders | SCB-2019 | 0 | 0 | 1 | 0 | 1 |
| Infections and infestations | ChAdOx1-S | 1 | 0 | 0 | 0 | 1 |
|  | SCB-2019 | 3 | 0 | 0 | 0 | 3 |
| Injury, poisoning and procedural complications | SCB-2019 | 2 | 0 | 0 | 0 | 2 |
| Musculoskeletal and connective tissue disorders | BNT162b2 | 1 | 0 | 0 | 0 | 1 |
|  | SCB-2019 | 1 | 0 | 0 | 0 | 1 |
| Nervous system disorders | BNT162b2 | 0 | 0 | 1 | 0 | 1 |
|  | SCB-2019 | 2 | 0 | 0 | 0 | 2 |
| Psychiatric disorders | BNT162b2 | 1 | 0 | 0 | 0 | 1 |
| Renal and urinary disorders | SCB-2019 | 1 | 0 | 0 | 0 | 1 |
| Respiratory, thoracic and mediastinal disorders | BNT162b2 | 1 | 0 | 0 | 0 | 1 |
